# Efficacy and safety of avacopan in patients aged 65 years and older with ANCA-associated vasculitis: a *post hoc* analysis of data from the ADVOCATE trial

**DOI:** 10.1093/rheumatology/keaf122

**Published:** 2025-03-03

**Authors:** Duvuru Geetha, Christian Pagnoux, Sebastian E Sattui, Peter A Merkel, Maria Weiner, Juliana Draibe, Stanislas Faguer, Sarah Bray, Rachel E Gurlin, Monica Balcells-Oliver, Annette Bruchfeld, David R Jayne, ADVOCATE Study Group, C Au Peh, A Chakera, B Cooper, J Kurtkoti, D Langguth, V Levidiotis, G Luxton, P Mount, D Mudge, E Noble, R Phoon, D Ranganathan, A Ritchie, J Ryan, M Suranyi, A Rosenkranz, K Lhotta, A Kronbichler, N Demoulin, C Bovy, R Hellemans, J Hougardy, B Sprangers, K Wissing, C Pagnoux, S Barbour, S Brachemi, S Cournoyer, L Girard, L Laurin, P Liang, D Philibert, M Walsh, V Tesar, R Becvar, P Horak, I Rychlik, W Szpirt, H Dieperink, J Gregersen, P Ivarsen, E Krarup, C Lyngsoe, C Rigothier, J Augusto, A Belot, D Chauveau, D Cornec, N Jourde-Chiche, M Ficheux, A Karras, A Klein, F Maurier, R Mesbah, O Moranne, A Neel, T Quemeneur, D Saadoun, B Terrier, P Zaoui, M Schaier, U Benck, R Bergner, M Busch, J Floege, F Grundmann, H Haller, M Haubitz, B Hellmich, J Henes, B Hohenstein, C Hugo, C Iking-Konert, F Arndt, T Kubacki, I Kotter, P Lamprecht, T Lindner, J Halbritter, H Mehling, U Schönermarck, N Venhoff, V Vielhauer, O Witzke, I Szombati, G Szucs, G Garibotto, F Alberici, E Brunetta, L Dagna, S De Vita, G Emmi, A Gabrielli, L Manenti, F Pieruzzi, D Roccatello, C Salvarani, M Harigai, H Dobashi, T Atsumi, S Fujimoto, N Hagino, A Ihata, S Kaname, Y Kaneko, A Katagiri, M Katayama, Y Kirino, K Kitagawa, A Komatsuda, H Kono, T Kurasawa, R Matsumura, T Mimura, A Morinobu, Y Murakawa, T Naniwa, T Nanki, N Ogawa, H Oshima, K Sada, E Sugiyama, T Takeuchi, H Taki, N Tamura, T Tsukamoto, K Yamagata, M Yamamura, P van Daele, A Rutgers, Y Teng, R Walker, I Chua, M Collins, K Rabindranath, J de Zoysa, M Svensson, B Grevbo, S Kalstad, M Little, M Clarkson, E Molloy, I Agraz Pamplona, J Anton, V Barrio Lucia, S Ciggaran, M Cinta Cid, M Diaz Encarnacion, X Fulladosa Oliveras, M Jose Soler, H Marco Rusinol, M Praga, L Quintana Porras, A Segarra, A Bruchfeld, M Segelmark, I Soveri, E Thomaidi, K Westman, T Neumann, M Burnier, T Daikeler, J Dudler, T Hauser, H Seeger, B Vogt, D Jayne, J Burton, R Al Jayyousi, T Amin, J Andrews, L Baines, P Brogan, B Dasgupta, T Doulton, O Flossmann, S Griffin, J Harper, L Harper, D Kidder, R Klocke, P Lanyon, R Luqmani, J McLaren, D Makanjuola, L McCann, A Nandagudi, S Selvan, E O'Riordan, M Patel, R Patel, C Pusey, R Rajakariar, J Robson, M Robson, A Salama, L Smyth, J Sznajd, J Taylor, P Merkel, A Sreih, E Belilos, A Bomback, J Carlin, Y Chang Chen Lin, V Derebail, S Dragoi, A Dua, L Forbess, D Geetha, P Gipson, R Gohh, G T Greenwood, S Hugenberg, R Jimenez, M Kaskas, T Kermani, A Kivitz, C Koening, C Langford, G Marder, A Mohamed, P Monach, N Neyra, G Niemer, J Niles, R Obi, C Owens, D Parks, A Podoll, B Rovin, R Sam, W Shergy, A Silva, U Specks, R Spiera, J Springer, C Striebich, A Swarup, S Thakar, A Tiliakos, Y Tsai, D Waguespack, M Chester Wasko

**Affiliations:** Division of Nephrology, Johns Hopkins University School of Medicine, Baltimore, MD, USA; Division of Rheumatology, Vasculitis Clinic, Mount Sinai Hospital, University of Toronto, Toronto, ON, Canada; Division of Rheumatology and Clinical Immunology, University of Pittsburgh, Pittsburgh, PA, USA; Division of Rheumatology, Department of Medicine, University of Pennsylvania, Philadelphia, PA, USA; Division of Epidemiology, Department of Biostatistics, Epidemiology, and Informatics, University of Pennsylvania, Philadelphia, PA, USA; Department of Nephrology and Health, Medicine and Caring Sciences, Linköping University, Linköping, Sweden; Department of Nephrology, Bellvitge University Hospital, Bellvitge Biomedical Research Institute (IDIBELL), Hospitalet de Llobregat, Barcelona, Spain; Department of Nephrology and Organ Transplantation, Toulouse University Hospital, Toulouse, France; Biostatistics, Amgen Ltd, Cambridge, UK; Medical Affairs, Amgen Inc, Thousand Oaks, CA, USA; Global Medical Affairs, CSL Vifor, Zurich, Switzerland; Department of Nephrology and Health, Medicine and Caring Sciences, Linköping University, Linköping, Sweden; Department of Renal Medicine, Karolinska University Hospital, CLINTEC Karolinska Institutet, Stockholm, Sweden; Department of Medicine, University of Cambridge, Cambridge, UK

**Keywords:** AAV, MPA, GPA, age, avacopan

## Abstract

**Objectives:**

To evaluate the efficacy and safety of avacopan in patients aged ≥65 years with granulomatosis with polyangiitis (GPA) or microscopic polyangiitis (MPA) in the phase 3 ADVOCATE trial of avacopan vs a prednisone taper, plus either rituximab or cyclophosphamide.

**Methods:**

In this descriptive, *post hoc* analysis, patients receiving avacopan or a prednisone taper were stratified by age. Key efficacy outcomes included the rate of remission at week 26 and sustained remission at week 52.

**Results:**

Of 160 patients aged ≥65, 109 were aged 65–74 and 51 were ≥75. Remission at week 26 was achieved in 71.7% vs 69.4% of patients aged 65–74 and 73.1% vs 72.0% aged ≥75 in the avacopan vs prednisone taper groups, respectively. Sustained remission at week 52 was observed in 65.0% vs 55.1% of patients aged 65–74 and 65.4% vs 56.0% aged ≥75. Relapse rates in the avacopan vs prednisone taper groups were 12.3% vs 18.8% and 3.8% vs 20.8% in the 65–74 and ≥75 subgroups, respectively. Improvements in estimated glomerular filtration rate and health-related quality of life were observed in both treatment groups. Use of avacopan compared with a prednisone taper was associated with a 61% and 49% reduction in mean glucocorticoid dose in the 65–74 and ≥75 subgroups, respectively, and lower glucocorticoid toxicity. The proportions of patients with adverse events were similar between treatment groups within each age subgroup.

**Conclusion:**

These data support the efficacy and safety of an avacopan-based regimen to treat patients with GPA or MPA aged ≥65.

Rheumatology key messages

- Patients with GPA or MPA aged ≥65 years have a high risk of glucocorticoid-related adverse effects and complications
- Avacopan showed similar efficacy and safety results in patients aged ≥65 years compared with those aged <65 years
- This analysis supports the use of an avacopan-based regimen in older patients with GPA or MPA to minimize glucocorticoid-related risks

## Introduction

Anti-neutrophil cytoplasmic antibody (ANCA)-associated vasculitis (AAV) is characterized by inflammation and damage of small- to medium-sized blood vessels [1]. The most common AAV subtypes are granulomatosis with polyangiitis (GPA) and microscopic polyangiitis (MPA) [1]. Although GPA and MPA have been described in all age groups, their incidence increases with age, with rates peaking in patients ≥65 years [2–4]. The disease type and pattern of organ involvement seen in patients with AAV differs with age [5, 6]. For example, patients ≥65 years of age at diagnosis display more kidney involvement and a higher risk of worsening kidney function compared with younger adults [6, 7]. Patients aged ≥65 years at diagnosis also present with higher rates of MPA and myeloperoxidase (MPO)-ANCA positivity compared with patients aged <65 years [6]. Compared with younger patients, older patients with AAV, especially those aged ≥75 years, tend to have more comorbidities, including hypertension, diabetes mellitus, heart disease and malignancy [8, 9] and a greater risk of death within the first 6 months after diagnosis of GPA or MPA [10, 11], mainly due to infection [11–14]. Generally, increased morbidity and mortality are seen in older patients (aged 66–90 years) with GPA or MPA compared with younger patients [8, 15]. The use of induction therapy has a survival benefit in older adults with AAV [8, 16]; however, differences in the treatment of older adults have been observed [11]. Results from one study suggested a lower risk of disease relapse in patients with GPA or MPA ≥75 years compared with patients aged 65–75 years [17].

Glucocorticoids (GCs) remain central to remission induction regimens for AAV [18, 19]. The relapsing and remitting nature of AAV means that patients are likely to experience repeated treatment courses and, therefore, high cumulative exposure to GCs [20]. The adverse effects of therapy with GCs are well documented and include infections, osteoporosis, cataracts, gastrointestinal effects, weight gain and diabetes mellitus [21–23]. Furthermore, GCs are major contributors to impaired quality of life [24, 25]. Advances in the treatment of AAV over the past 20 years, including the use of rituximab and combination therapies, have improved patient prognosis [24, 26, 27]. Clinical trials and observational studies demonstrated the success of low-dose GC protocols in achieving remission [28–30]. However, some studies excluded patients aged over 75 or 80 years [8, 31], resulting in an evidence gap in this population at high risk of disease- and treatment-related morbidity and mortality. Minimization of the use of GCs to avoid the toxicity associated with these agents is a focus of treatment [32]. While a low-dose GC regimen seems favourable for the treatment of patients with GPA or MPA aged ≥65 years in terms of reduced rates of infection [33–35], improved survival [16] and lower rates of serious adverse events (SAEs) [36], older adults are commonly underrepresented in clinical trials.

Avacopan is an orally administered small-molecule complement C5a receptor (C5aR1) antagonist that selectively blocks the effects of C5a through the C5aR1, including blocking neutrophil chemoattraction and activation [37]. In the phase 3 ADVOCATE trial of avacopan, there was no exclusion criterion for maximum participant age, presenting an opportunity to investigate disease presentation and treatment response in a clinical trial among patients aged ≥65 years [37]. The aim of this analysis was to evaluate the efficacy and safety of avacopan compared with a prednisone taper in three subgroups of patients stratified by age (<65 years, 65–74 years and ≥75 years) in the ADVOCATE trial, with a focus on those aged ≥65 years.

## Methods

### Study design and participants

ADVOCATE was a 52-week, double-blind, randomized, active-controlled trial in patients with GPA or MPA [37]. The study design and complete inclusion and exclusion criteria of ADVOCATE (NCT02994927) have been described previously [37]. 331 patients were enrolled across 143 centres and randomized to receive 30 mg of avacopan twice daily orally plus prednisone-matching placebo, or a tapering oral regimen of prednisone plus avacopan-matching placebo in a double-dummy design. Patients were randomly assigned in a 1:1 ratio to treatment groups and stratified by disease status (newly diagnosed vs relapsing), ANCA status (anti-proteinase 3 [PR3] vs anti-MPO) and standard-of-care induction treatment (rituximab or cyclophosphamide), which all patients received. The trial was performed in accordance with the principles of the Declaration of Helsinki and Good Clinical Practice guidelines. Ethics committees and institutional review boards at participating sites approved the research protocol. All patients gave written informed consent before study entry.

### Outcomes and analysis

This analysis reports on the efficacy and safety of avacopan compared with a prednisone taper in the modified intention-to-treat population in subgroups of patients aged <65 years (*n* = 170), 65–74 years (*n* = 109) and ≥75 years (*n* = 51). Baseline characteristics, including disease status and presentation, were assessed. Remission was defined by a BVAS of 0 and no GC use for vasculitis in the preceding 4 weeks. The key efficacy outcomes evaluated were rate of remission at week 26 and sustained remission at week 52. Patients were not considered to be in sustained remission if they had remission at week 26 but a relapse thereafter; relapse was defined as a return of vasculitis activity based on BVAS with one or two minor items for at least two consecutive trial visits, ≥1 major item, or ≥3 minor items. An exploratory outcome was the rate of relapse at any time after a BVAS of 0 was first achieved. Kidney function was assessed by least squares mean (LSM) change from baseline in estimated glomerular filtration rate (eGFR) in patients with kidney involvement (based on BVAS) and an early change in urinary albumin-to-creatinine ratio (UACR) from baseline to week 4 in patients with kidney involvement (based on BVAS) and a UACR ≥10 mg/g creatinine at baseline. Total all-source GC dose, Glucocorticoid Toxicity Index (GTI) Cumulative Worsening Score (CWS) and GTI Aggregate Improvement Score (AIS) were evaluated. Higher GTI-CWS and GTI-AIS indicate more severe toxic effects. Health-related quality of life (HRQoL) was assessed using the Medical Outcomes Study Short Form 36 (SF-36) version 2 physical and mental component summary scores (PCS and MCS) and the EuroQol group 5-Dimension 5-Level (EQ-5D-5L) index score and visual analogue scale (VAS). For all HRQoL instruments, higher scores indicate better quality of life. Safety outcomes included the occurrence of all adverse events (AEs), including those possibly related to GCs or infection.

### Statistical analysis

This study was a descriptive analysis reporting means and 95% CIs. The key efficacy analyses were conducted in the modified intention-to-treat population, defined as all randomly assigned patients who received at least one dose of trial medication. Summary score estimates of the common difference in the incidences of remission were calculated with the use of inverse-variance stratum weights. Missing end point data at week 26 and week 52 were imputed as no remission. Data were analysed after all patients had completed the 52-week treatment period, and no interim analyses were performed. LSMs, standard errors and CIs were calculated using models incorporating treatment group, visit, treatment-by-visit interaction and stratification factors as covariates. Longitudinal measurements from the same patients were considered as repeated-measure units in the model. The trial was not powered to draw definitive conclusions from these subgroup analyses.

## Results

### Baseline demographics, characteristics and comorbidities

Baseline demographics and clinical characteristics of patients treated with avacopan were generally similar to those treated with prednisone taper within all age subgroups (Table 1). There was a trend of mean baseline body mass index (BMI) decreasing in the older age groups, with the lowest mean baseline BMI in the ≥75 years subgroup. MPO-ANCA positivity at baseline was more frequent in the 65–74 years and ≥75 years subgroups. Relapsing disease at baseline was more frequent in the <65 years subgroup. The frequency of comorbidities at baseline was higher in the 65–74 and ≥75 years subgroups than in the <65 years subgroup for the majority of comorbidities (Supplementary Table S1, available at *Rheumatology* online). By system organ class, the most frequently occurring comorbidities in the <65 years subgroup were respiratory, thoracic and mediastinal disorders, occurring in 108 (63.5%) patients. In the 65–74 and ≥75 years subgroups, vascular disorders were the most frequently occurring comorbidities, present in 82 (75.2%) and 37 (72.5%) patients, respectively.

**Table 1.** Baseline characteristics of patients with GPA or MPA in the ADVOCATE trial of avacopan, stratified by age

Baseline characteristic	Age <65 years			Age 65–74 years			Age ≥75 years		
	Avacopan (*n* = 80)	Prednisone taper (*n* = 90)	Total (*n* = 170)	Avacopan (*n* = 60)	Prednisone taper (*n* = 49)	Total (*n* = 109)	Avacopan (*n* = 26)	Prednisone taper (*n* = 25)	Total (*n* = 51)
Sex, *n* (%)									
Male	53 (66.3)	54 (60.0)	107 (62.9)	32 (53.3)	22 (44.9)	54 (49.5)	13 (50.0)	12 (48.0)	25 (49.0)
Female	27 (33.8)	36 (40.0)	63 (37.1)	28 (46.7)	27 (55.1)	55 (50.5)	13 (50.0)	13 (52.0)	26 (51.0)
Race, *n* (%)									
Asian	3 (3.8)	6 (6.7)	9 (5.3)	5 (8.3)	3 (6.1)	8 (7.3)	9 (34.6)	6 (24.0)	15 (29.4)
White	69 (86.3)	77 (85.6)	146 (85.9)	52 (86.7)	44 (89.8)	96 (88.1)	17 (65.4)	19 (76.0)	36 (70.6)
Other^a^	8 (10)	7 (7.8)	15 (8.8)	3 (5.0)	2 (4.1)	5 (4.6)	0 (0.0)	0 (0.0)	0 (0.0)
BMI (kg/m^2^), mean (s.d.)	27.2 (6.2)	27.6 (5.3)	27.4 (5.7)	27.1 (6.3)	26.6 (4.7)	26.8 (5.6)	24.6 (3.9)	24.4 (5.2)	24.5 (4.6)
Type of AAV, *n* (%)									
GPA	48 (60.0)	60 (66.7)	108 (63.5)	33 (55.0)	22 (44.9)	55 (50.5)	10 (38.5)	8 (32.0)	18 (35.3)
MPA	32 (40.0)	30 (33.3)	62 (36.5)	27 (45.0)	27 (55.1)	54 (49.5)	16 (61.5)	17 (68.0)	33 (64.7)
AAV status, *n* (%)									
Newly diagnosed	49 (61.3)	56 (62.2)	105 (61.8)	45 (75.0)	38 (77.6)	83 (76.1)	21 (80.8)	20 (80.0)	41 (80.4)
Relapsed	31 (38.8)	34 (37.8)	65 (38.2)	15 (25.0)	11 (22.4)	26 (23.9)	5 (19.2)	5 (20.0)	10 (19.6)
ANCA positivity, *n* (%)									
PR3-ANCA	43 (53.8)	53 (58.9)	96 (56.5)	22 (36.7)	15 (30.6)	37 (33.9)	7 (26.9)	2 (8.0)	9 (17.6)
MPO-ANCA	37 (46.3)	37 (41.1)	74 (43.5)	38 (63.3)	34 (69.4)	72 (66.1)	19 (73.1)	23 (92.0)	42 (82.4)
Duration of AAV (months), mean (s.d.)	32.0 (67.3)	26.0 (45.9)	28.8 (56.9)	16.5 (34.9)	12.5 (26.4)	14.7 (31.3)	9.7 (20.5)	14.0 (40.4)	11.8 (31.6)
Standard-of-care treatment, *n* (%)									
IV rituximab	57 (71.3)	57 (63.3)	114 (67.1)	34 (56.7)	34 (69.4)	68 (62.4)	16 (61.5)	16 (64.0)	32 (62.7)
IV cyclophosphamide	20 (25.0)	31 (34.4)	51 (30.0)	22 (36.7)	12 (24.5)	34 (31.2)	9 (34.6)	8 (32.0)	17 (33.3)
Oral cyclophosphamide	3 (3.8)	2 (2.2)	5 (2.9)	4 (6.7)	3 (6.1)	7 (6.4)	1 (3.8)	1 (4.0)	2 (3.9)
BVAS score, mean (s.d.)	15.6 (5.7)	15.3 (5.5)	15.5 (5.6)	16.6 (5.5)	16.5 (5.9)	16.6 (5.7)	17.8 (7.1)	18.4 (5.6)	18.1 (6.3)
VDI score, mean (s.d.)	0.6 (1.3)	0.8 (1.4)	0.7 (1.3)	0.8 (1.9)	0.6 (1.3)	0.7 (1.7)	0.6 (1.4)	0.5 (1.7)	0.6 (1.5)
Kidney disease at baseline, *n* (%)	65 (81.3)	68 (75.6)	133 (78.2)	49 (81.7)	43 (87.8)	92 (84.4)	20 (76.9)	23 (92.0)	43 (84.3)
eGFR (mL/min/1.73 m^2^), mean (s.d.)^b^	53.9 (31.1)	53.1 (31.8)	53.5 (31.5)	40.2 (22.7)	41.5 (19.2)	40.8 (21.1)	25.9 (10.3)	30.8 (16.5)	28.5 (14.0)
UACR (mg/g), geometric mean^c^	495.7	347.3	417.4	393.4	290.6	346.2	360.0	258.1	305.3

Data were missing for two patients for BMI, two patients for VDI and four patients for eGFR. UACR data were missing for 77 patients.

a Other race included Black or African American, multiple or other races.

b eGFR is reported in patients with kidney disease (based on BVAS) at baseline.

c UACR is reported in patients with kidney disease at baseline (based on BVAS) and albuminuria (UACR ≥10 mg/g creatinine).

AAV: ANCA-associated vasculitis; eGFR: estimated glomerular filtration rate; GPA: granulomatosis with polyangiitis; IV: intravenous; MPA: microscopic polyangiitis; MPO: myeloperoxidase; PR3: proteinase 3; UACR: urine albumin-to-creatinine ratio; VDI: Vasculitis Damage Index.

### Remission and relapse rates

When evaluated by age, the rates of remission were similar to the overall trial results [37]. Across all age subgroups and treatment groups, between 69.4% and 73.1% of patients achieved remission at week 26 (Table 2). Sustained remission at week 52 was observed in 54.4–66.3% of patients across all age subgroups, with a higher percentage achieved in the avacopan group within each age subgroup (percentage difference between treatment groups: <65 years, 11.8% [95% CI: −2.8, 26.4]; 65–74 years, 9.9% [95% CI: −8.5, 28.3]; ≥75 years, 9.4% [95% CI: −17.3, 36.1]). Relapse rates in the avacopan vs prednisone taper groups were 12.3% vs 18.8% and 3.8% vs 20.8% in the 65–74 years and ≥75 years subgroups, respectively.

**Table 2.** Key efficacy endpoints in patients with GPA or MPA in the ADVOCATE trial of avacopan, stratified by age

Outcome	Age <65 years (*n* = 170)		Age 65–74 years (*n* = 109)		Age ≥75 years (*n* = 51)	
	Avacopan (*n* = 80)	Prednisone taper (*n* = 90)	Avacopan (*n* = 60)	Prednisone taper (*n* = 49)	Avacopan (*n* = 26)	Prednisone taper (*n* = 25)
Remission at week 26, *n* (%)	58 (72.5)	63 (70.0)	43 (71.7)	34 (69.4)	19 (73.1)	18 (72.0)
Difference between treatment groups (%) [two-sided 95% CI]	2.5 [−11.1, 16.1]		2.3 [−14.9, 19.5]		1.1 [−23.4, 25.6]	
Sustained remission at week 52, *n* (%)	53 (66.3)	49 (54.4)	39 (65.0)	27 (55.1)	17 (65.4)	14 (56.0)
Difference between treatment groups (%) [two-sided 95% CI]	11.8 [−2.8, 26.4]		9.9 [−8.5, 28.3]		9.4 [−17.3, 36.1]	
Relapse rate,^a^ *n* (%)	8 (10.7)	19 (22.4)	7 (12.3)	9 (18.8)	1 (3.8)	5 (20.8)
Hazard ratio [95% CI]	0.52 [0.18, 1.53]		0.64 [0.24, 1.72]		0.18 [0.02, 1.53]	
UACR percent change at week 4,^b^ LSM ± s.e.m.	−45 ± 14	15 ± 14	−34 ± 16	−19 ± 17	−33 ± 26	−8 ± 25
LSM difference [95% CI]	−52 [−67, −31]		−19 [−47, 23]		−28 [−62, 37]	
GTI-CWS at week 26, LSM ± s.e.m.	40.2 ± 4.8	60.3 ± 4.4	43.4 ± 6.3	53.6 ± 7.2	33.1 ± 8.8	51.4 ± 10.1
LSM difference [95% CI]	−20.1 [−32.0, −8.1]		−10.3 [−27.2, 6.7]		−18.3 [−40.3, 3.7]	
GTI-AIS at week 26, LSM ± s.e.m.	13.7 ± 5.1	30.5 ± 4.6	13.6 ± 5.9	15.1 ± 6.8	0.4 ± 8.5	15.1 ± 9.7
LSM difference [95% CI]	−16.9 [−29.6, −4.2]		−1.5 [−17.6, 14.6]		−14.7 [−35.6, 6.2]	
Total all-source GC dose, mg (mean/median)	1861/620	4122/3230	1410/575	3579/3055	1718/588	3382/2840

a Relapse rates are based on the number of patients who achieved a BVAS of 0 after any time during the 52-week treatment period.

b UACR is reported in patients with kidney involvement (based on BVAS at baseline) and baseline UACR ≥10 mg/g creatinine.

AIS: Aggregate Improvement Score; CWS: Cumulative Worsening Score; GC: glucocorticoid; GPA: granulomatosis with polyangiitis; GTI: Glucocorticoid Toxicity Index; LSM: least squares mean; MPA: microscopic polyangiitis; UACR: urine albumin-to-creatinine ratio.

### Change in eGFR and UACR

**Figure 1.**
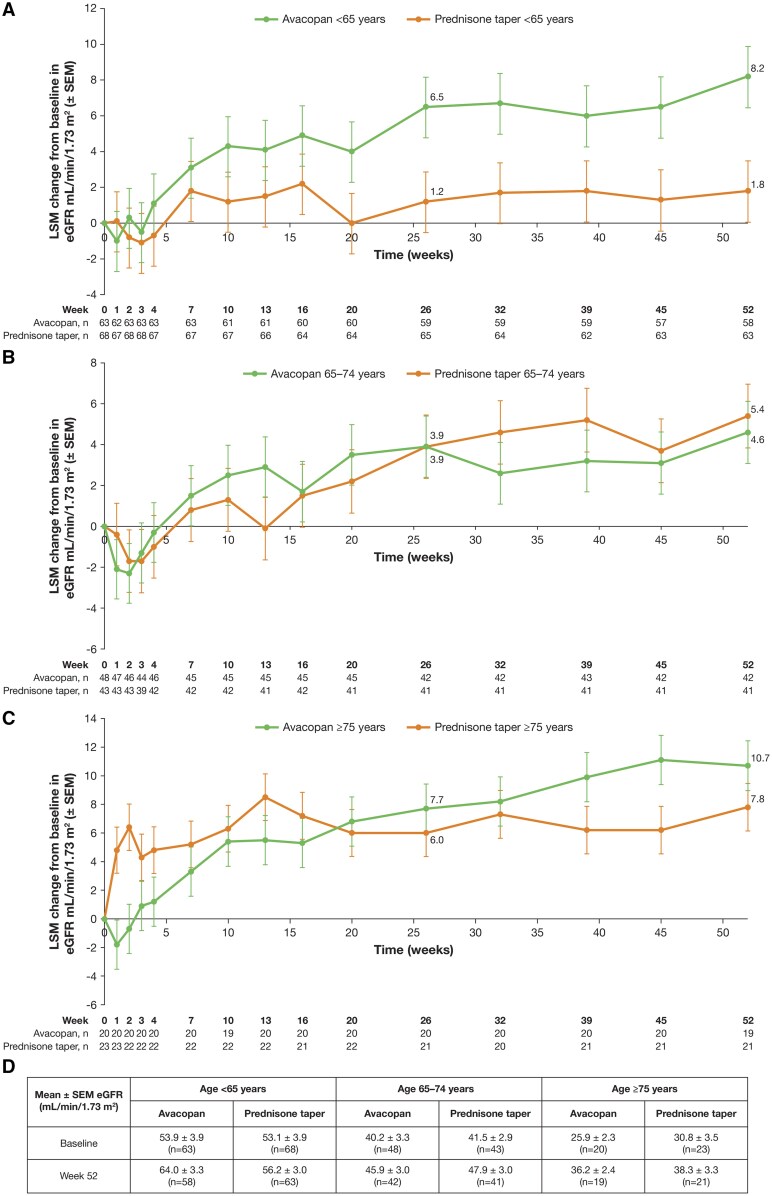
eGFR during a 52-week treatment period in patients with GPA or MPA in the ADVOCATE trial. (A–C) Change in eGFR from baseline in patients aged <65, 65–74 and ≥75 years, respectively. (D) Mean ± s.e.m. eGFR at baseline and week 52 in patients aged <65, 65–74 and ≥75 years. eGFR was assessed only in patients with kidney involvement (based on BVAS) at baseline. eGFR: estimated glomerular filtration rate; LSM: least squares mean; MPA: microscopic polyangiitis

Baseline eGFR in patients with kidney disease (based on BVAS) was lower in the older age subgroups (Table 1). Change in eGFR increased over the 52-week study period in every age subgroup and both treatment groups (Fig. 1). The largest LSM change from baseline was seen in patients ≥75 years treated with avacopan at week 52: 10.7 ± 1.7 mL/min/1.73 m^2^ (baseline eGFR: 25.9 ± 10.3 mL/min/1.73 m^2^) vs 7.8 ± 1.7 mL/min/1.73 m^2^ with a prednisone taper (baseline eGFR: 30.8 ± 16.5 mL/min/1.73 m^2^) (LSM difference: 2.9; 95% CI: −1.8, 7.7) (Fig. 1C). The LSM difference at week 52 in the 65–74 years subgroup was −0.8; 95% CI −5.1, 3.4 and 6.4; 95% CI: 1.6, 11.1 in the <65 years subgroup. The early reduction in UACR observed at week 4 in patients with kidney involvement (based on BVAS) and a UACR ≥10 mg/g creatinine in the avacopan group is shown in Table 2.

### GC dose and toxicity

The total all-source median GC dose at week 52 was approximately five-fold higher in the prednisone taper group than in the avacopan group within all three age subgroups (Table 2). Total all-source mean GC dose from day 0 to week 52 was decreased in the avacopan group by 55%, 61% and 49% for the <65 years, 65–74 years and ≥75 years subgroups, respectively. GTI-CWS was also higher in the prednisone taper group than in the avacopan group across all age subgroups (Table 2), with an LSM (95% CI) difference between groups (avacopan vs prednisone taper) of −20.1 (−32.0, −8.1) in the <65 years age subgroup, −10.3 (−27.2, 6.7) in the 65–74 years subgroup and −18.3 (−40.3, 3.7) in the ≥75 years subgroup. GTI-AIS (LSM ± standard error of the mean) at week 26 was particularly low with avacopan (0.4 ± 8.5) compared with a prednisone taper (15.1 ± 9.7) in the ≥75 years subgroup. LSM (95% CI) difference between treatment groups in GTI-AIS was −16.9 (−29.6, −4.2), −1.5 (−17.6, 14.6) and −14.7 (−35.6, 6.2) in the <65 years, 65–74 years and ≥75 years subgroups, respectively.

### HRQoL outcomes

**Figure 2.**
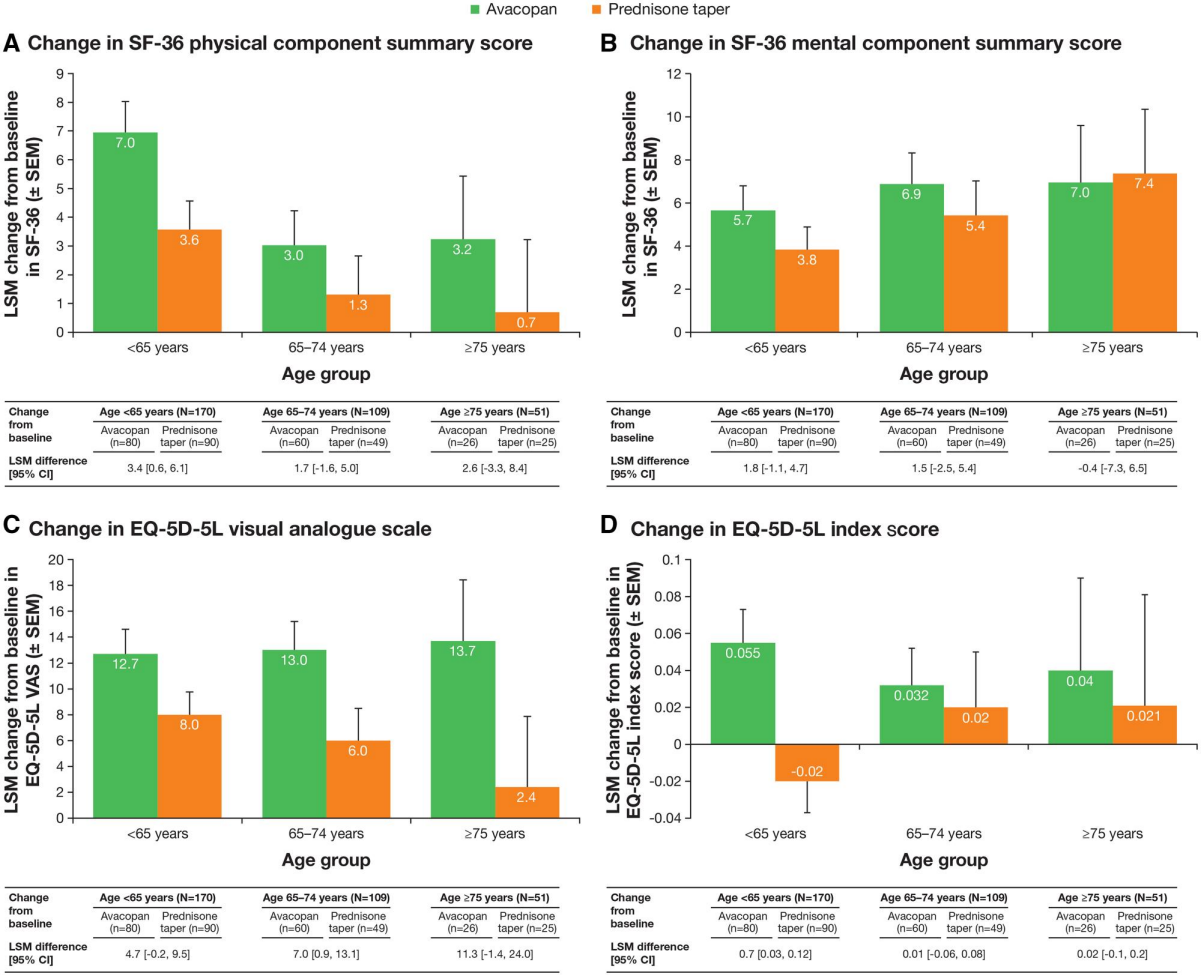
Health-related quality of life outcomes of (A) SF-36 physical component summary score, (B) SF-36 mental component summary score, (C) EQ-5D-5L VAS and (D) EQ-5D-5L index score at week 52 in patients with GPA or MPA stratified by age treated with avacopan or a prednisone taper. EQ-5D-5L: EuroQol 5-Dimension 5-Level; GPA: granulomatosis with polyangiitis; LSM: least squares mean; MPA: microscopic polyangiitis; SF-36: Short Form-36; VAS: visual analogue scale

Results for HRQoL outcomes at week 52, as measured by the SF-36 PCS and MCS scores, EQ-5D-5L VAS and EQ-5D-5L index score are shown in Fig. 2. At week 52, change from baseline in HRQoL was generally higher in the avacopan group than in the prednisone taper group across most age subgroups, except the SF-36 MCS in the ≥75 years subgroup.

### Safety outcomes

The proportions of AEs and SAEs were broadly similar between treatment groups within each age subgroup (Table 3). In the avacopan and prednisone taper groups, respectively, hepatic enzymes increased in 1 (1.3%) vs 5 (5.6%) patients aged <65 years, 1 (1.7%) vs 1 (2%) patients aged 65–74 years, and 3 (11.5%) vs 1 (4.0%) patients aged ≥75 years; cardiac disorders occurred in 9 (11.3%) vs 7 (7.8%) patients in the <65 years subgroup, 13 (21.7%) vs 8 (16.3%) patients in the 65–74 years subgroup and 4 (15.4%) vs 6 (24%) patients in the ≥75 years subgroup; and neoplasms benign, malignant and unspecified (including cysts and polyps) occurred in 3 (3.8%) vs 7 (7.8%) patients aged <65 years, 1 (1.7%) vs 7 (14.3%) patients aged 65–74 years and 2 (7.7%) vs 2 (8%) patients aged ≥75 years.

**Table 3.** Safety results in patients with GPA or MPA treated with avacopan or prednisone taper, stratified by age

Outcome	Age <65 years (*n* = 170)		Age 65–74 years (*n* = 109)		Age ≥75 years (*n* = 51)	
	Avacopan (*n* = 80)	Prednisone taper (*n* = 90)	Avacopan (*n* = 60)	Prednisone taper (*n* = 49)	Avacopan (*n* = 26)	Prednisone taper (*n* = 25)
Total AEs, *n* (%) patients, *n* events	79 (98.8) 883 events	88 (97.8) 1140 events	59 (98.3) 623 events	48 (98.0) 681 events	26 (100.0) 273 events	25 (100.0) 318 events
AEs possibly related to GCs, *n* (%) patients, *n* events	53 (66.3) 198 events	70 (77.8) 269 events	30 (50.0) 124 events	41 (83.7) 164 events	24 (92.3) 73 events	20 (80.0) 105 events
Any AE related to infection, *n* (%) patients, *n* events	53 (66.3) 105 events	66 (73.3) 163 events	40 (66.7) 86 events	38 (77.6) 88 events	20 (76.9) 42 events	20 (80.0) 40 events
Total SAEs, *n* (%) patients, *n* events	28 (35.0) 51 events	38 (42.2) 81 events	25 (41.7) 43 events	22 (44.9) 51 events	17 (65.4) 22 events	14 (56.0) 34 events
SAEs possibly related to GCs, *n* (%) patients, *n* events	4 (5.0) 5 events	16 (17.8) 18 events	7 (11.7) 8 events	4 (8.2) 7 events	5 (19.2) 5 events	7 (28.0) 11 events
Any SAE related to infection, *n* (%) patients, *n* events	7 (8.8) 8 events	14 (15.6) 17 events	11 (18.3) 13 events	5 (10.2) 6 events	4 (15.4) 4 events	6 (24.0) 8 events
Hospitalization possibly related to study medication, *n* (%) patients, *n* events	4 (5.0) 5 events	11 (12.2) 13 events	5 (8.3) 6 events	4 (8.2) 8 events	5 (19.2) 5 events	5 (20.0) 7 events
Deaths, *n* (%) patients	0 (0.0)	2 (2.2)	2 (3.3)	2 (4.1)	0 (0.0)	0 (0.0)

AE: adverse event; GC: glucocorticoid; SAE: serious adverse event.

## Discussion

This *post hoc* subgroup analysis from the ADVOCATE trial demonstrates that avacopan when added to standard-of-care immunosuppression, is equally effective compared with a prednisone taper for the treatment of GPA or MPA with respect to remission at week 26, and more effective for sustained remission at week 52 in patients aged ≥65 years. Avacopan was also associated with a greater improvement in HRQoL compared with a prednisone taper in patients aged ≥65 years. These results are consistent with those from the <65 years subgroup. In addition, the proportion of AEs and SAEs was broadly similar between treatment groups within the 65–74 years and ≥75 years subgroups, as in the <65 years subgroup. The addition of avacopan to the standard of care in the treatment of older adults (≥65 years) with GPA or MPA is associated with efficacy and safety similar to that observed in younger adults and should be considered in this older population, who remain at higher risk of poor outcomes.

Efficacy and safety outcomes in the avacopan group of the 65–74 years and ≥75 years subgroups were achieved with an ∼80% reduction in median overall total GC dose compared with the prednisone taper group. The marked reduction in the use of GCs in the avacopan treatment group was per the study protocol and is among the key benefits of the avacopan-based treatment regimen. This outcome would likely be particularly beneficial for older patients, who are more susceptible to adverse effects of GCs [36]. Notably, the GTI-AIS was reduced with avacopan compared with a prednisone taper in the ≥75 years subgroup.

A high proportion of patients aged 65–74 years and ≥75 years in this study presented with kidney involvement (based on BVAS) at baseline (∼84%), and among those patients, mean baseline eGFR was lower in the ≥75 years subgroup compared with the 65–74 years and the <65 years subgroups. This observation is consistent with previous studies showing that GPA or MPA is more likely to present with more serious kidney involvement in patients aged ≥65 years than in younger patients [6, 7]. This analysis demonstrates that avacopan is associated with improved kidney function in patients aged ≥75 years. However, it is important to note that the difference between treatment groups in change in eGFR throughout treatment was larger in the <65 years subgroup than in the two older subpopulations. Reasons for this may include age-related decline in eGFR, higher rates of comorbidities at baseline, or a higher proportion of MPO-ANCA positivity in older patients than in the <65 years subgroup, which may indicate increased presence of subclinical disease during years before diagnosis and kidney fibrosis. These characteristics may also contribute to a low eGFR at baseline, worse kidney recovery and persistence of elevated UACR after treatment.

AAV is associated with reductions in HRQoL [38–42]. In the current analysis, the change in EQ-5D-5L VAS score from baseline to the end of treatment was larger in the avacopan vs prednisone taper group in all age subgroups (Fig. 2), as was previously reported in the analysis of HRQoL in the ADVOCATE trial [42]. The LSM difference between treatment groups at week 52 was largest in the ≥75 years subgroup for SF-36 PCS score and EQ-5D-5L VAS. These data show that incorporation of avacopan into the management strategies of patients with GPA or MPA aged ≥65 years could improve the quality of life in these patients.

Avacopan is included in the Kidney Disease Improving Global Outcomes (KDIGO) 2024 clinical practice guideline for the management of AAV and the 2022 European Alliance of Associations for Rheumatology (EULAR) recommendations for the management of GPA or MPA [19, 43]. The KDIGO guideline states that ‘avacopan may be used as an alternative to glucocorticoids. Patients with an increased risk of glucocorticoids toxicity are likely to receive the most benefit from avacopan. Patients with lower GFR may benefit from greater GFR recovery’ [43]. According to the EULAR recommendations, avacopan, used in combination with rituximab or cyclophosphamide, may be considered for induction of remission in GPA or MPA as part of a strategy to substantially reduce exposure to GCs. These recommendations include implementing individualized treatment strategies based on the patient’s disease course, presentation and comorbidities [19]. Clinicians could consider incorporating avacopan into the treatment regimens of patients at the highest risk of GC-related adverse effects and complications (such as osteoporosis, cataracts and GC-induced diabetes mellitus [21–23, 44]) or patients with severe kidney involvement. Emerging data regarding relevant risk factors (e.g. frailty) in this age group will better inform treatment in older adults and personalize the management of these patients [11, 45]. Based on the results from this analysis, avacopan may play a role in defining patient-tailored therapeutic strategies in patients with GPA or MPA aged ≥65 years at high risk of GC-related toxicity.

This analysis has several strengths, including the inclusion of data derived from a randomized trial and the use of defined outcomes of importance to clinicians and patients. This analysis also demonstrated the importance and value of including older patients in clinical trials. The limitations of this study include the relatively small sample size of the subpopulations discussed, increasing the probability of a type I error and potentially limiting the ability to generalize the findings, and the *post hoc* nature of the analysis. The conclusions based on kidney involvement are limited because the eligibility criteria from the original trial excluded patients with eGFR <15 mL/min/1.73 m^2^.

## Conclusions

This analysis from the ADVOCATE trial of patients stratified by age demonstrated similar trends of efficacy and safety of avacopan in patients aged ≥65 years as in patients aged <65 years, including improvements in measures of HRQoL and reductions in GC-related toxicities. These results highlight the efficacy and safety of an avacopan-based regimen, with minimization of GC use, in addition to rituximab or cyclophosphamide, for induction of remission in patients with GPA or MPA aged ≥65 years.

## Supplementary Material

keaf122_Supplementary_Data

## Data Availability

Data are available on reasonable request from qualified external scientific and medical researchers and based on an appropriate scientific rationale/analysis plan. Please contact phase4@viforpharma.com.
