# Supplementary material for: Efficacy and safety of avacopan in patients aged 65 years and older with ANCA-associated vasculitis: a post hoc analysis of data from the ADVOCATE trial
Source: Rheumatology (Oxford). 2025 Mar 3;64(6):3863–71. doi: 10.1093/rheumatology/keaf122 (PMC12107037; doi:10.1093/rheumatology/keaf122)
Supplement: keaf122_Supplementary_Data [file keaf122_supplementary_data.zip › keaf122_Supplementary_Data/rhe-24-2844-File004.pdf]

ADVOCATE PARTICIPATING INVESTIGATORS

(Presented by country, with National Coordinating Center followed by participating centers, in alphabetical order by principal investigator.)

Australia— National Coordinating Center: Royal Adelaide Hospital, Adelaide SA (C. Au Peh); Sir Charles Gairdner Hospital, Nedlands, WA (A. Chakera); Royal North Shore Hospital, St Leonards (B. Cooper); Griffith University, Southport (J. Kurtkoti); Wesley Medical Research, Auchenflower (D. Langguth); Western Health, St. Albans Victoria (V. Levidiotis); Prince of Wales Hospital, Randwick NSW (G. Luxton); Austin Health, Heidelberg Victoria (P. Mount); Princess Alexandra Hospital, Woolloongabba, QLD (D. Mudge); Sunshine Coast University Hospital, Birtinya (E. Noble); Westmead Hospital, Westmead NSW (R. Phoon); Royal Brisbane and Women's Hospital, Herston QLD (D. Ranganathan); Concord Repatriation General Hospital, Concord (A. Ritchie); Monash Medical Centre, Clayton Victoria (J. Ryan); Liverpool Hospital, Liverpool, NSW (M. Suranyi).

Austria— National Coordinating Center: Medizinische Universitaet Graz, Graz (A. Rosenkranz); Landeskrankenhaus Feldkirch, Feldkirch (K. Lhotta); Medical University of Innsbruck, Innsbruck (A. Kronbichler);

Belgium— National Coordinating Center: Cliniques Universitaires Saint-Luc, Brussels (N. Demoulin); Centre Hospitalier Universitaire (CHU) de Liege, Liege (C. Bovy); Antwerp University Hospital (UZA), Edegem (R. Hellemans); Universite Libre de Bruxelles (ULB) - Hopital Erasme, Brussels (J. Hougardy); University Hospital (UZ) Leuven, Leuven (B. Sprangers); University Hospital Brussels, Brussels (K. Wissing);.

Canada— National Coordinating Center: University of Toronto, Toronto (C. Pagnoux); St. Paul Hospital, Vancouver (S. Barbour); Centre de Recherche du Centre Hospitalier de l'Université de Montréal, Montreal (S. Brachemi); CISSS de la Monteregie-Centre – Hopital Charles LeMoyne, Greenfield Park (S. Cournoyer); University of Calgary, Calgary (L. Girard); Hospital Maisonneuve-Rosemont, Montreal (L. Laurin); Centre Hospitalier Universitaire de Sherbrooke, Sherbrooke (P. Liang); CHUQ-L'Hotel-Dieu de Quebec, Quebec City (D. Philibert); St. Josephs Healthcare, Hamilton (M. Walsh).

Czech Republic— Department of Nephrology, General University Hospital, Prague (V. Tesar); Rheumatology Institute, Prague (R. Becvar); University Hospital Olomouc, Olomouc (P. Horak); University Hospital Vinohrady, Prague (I. Rychlik).

Denmark— National Coordinating Center: Copenhagen University Hospital, Copenhagen (W. Szpirt); Odense University Hospital, Odense (H. Dieperink); Aalborg University Hospital, Aalborg (J. Gregersen); Aarhus University Hospital - Skejby, Aarhus (P. Ivarsen); Herlev Hospital, Herlev (E. Krarup); Sjaellands Universitetshospital Roskilde, Roskilde (C. Lyngsoe).

France— National Coordinating Center: CHU Bordeaux - Hospital Pellegrin, Bordeaux (C. Rigothier); CHU Angers, Angers (J. Augusto); CHU Lyon- Hopital Femme- Mere-Enfant, Bron (A. Belot); CHU de Toulouse - Hospital Rangueil, Toulouse (D. Chauveau); CHU de Brest - Hopital de la Cavale Blanche, Brest (D. Cornec); APHM - Hopital de la Conception, Marseille (N. Jourde-Chiche); CHU de Caen, Caen (M. Ficheux); Hopital Europeen Georges Pompidou, Paris (A. Karras); Hopitaux Civils de Colmar, Colmar (A. Klein); Hopitaux Prives de Metz, Metz (F. Maurier); Centre Hospitalier Boulogne sur Mer, Boulogne sur Mer (R. Mesbah); CHU Nimes – Hopital Caremeau, Nimes (O. Moranne); CHU Nantes Medicine Interne, Nantes (A. Neel); Centre Hospitalier de Valenciennes, Valenciennes (T. Quemeneur); Hospital Pitie Salpetriere, Paris (D. Saadoun); Hopital Cochin, Paris (B. Terrier); CHU de Grenoble, Grenoble Isere Cedex (P. Zaoui).

Germany— National Coordinating Center: University Clinic Heidelberg, Heidelberg (M. Schaier); University Clinic Mannheim, Mannheim (U. Benck); Clinic of Ludwigshafen am Rhein, Ludwigshafen (R. Bergner); University Clinic Jena, Jena (M. Busch); University Clinic Aachen, Aachen (J. Floege); University Clinic Cologne, Cologne (F. Grundmann); Medizinische Hochschule Hannover, Hannover (H. Haller); Klinikum Fulda, Fulda (M. Haubitz); Medius Clinic Kirchheim, Kirchheim-unter-Teck (B. Hellmich); University Hospital Tuebingen, Tuebingen (J. Henes); Nephrological Center Villingen-Schwenningen, Villingen-Schwenningen (B. Hohenstein); University Clinic Carl Gustav Carus, Dresden (C. Hugo); Klinikum Bad Bramstedt GmbH, Bad Bramstedt (C. Iking-Konert and F. Arndt); Asklepios Kinik, Hamburg (T. Kubacki and I. Kotter); University Clinic Schleswig-Holstein, Luebeck (P. Lamprecht); University Clinic Leipzig, Leipzig (T. Lindner and J. Halbritter); Charité - Universitaetsmedizin Berlin, Berlin (H. Mehling); Universität München – Großhadern, Munich (U. Schönermarck); University Clinic Freiburg, Freiburg (N. Venhoff); University Clinic Munich, Munich (V. Vielhauer); University Clinic Essen, Essen (O. Witzke).

Hungary— Qualiclinic Kft, Budapest (I. Szombati); DEOEC Rheumatology Faculty, Debrecen (G. Szucs).

Italy— National Coordinating Center: IRCCS Azienda Ospedaliera Universitaria San Martino, Genova (G. Garibotto); ASST Santi Paolo e Carlo-Presidio Ospedale San Carlo, Milan (F. Alberici); Istituto Clinico Humanitas, Rozzano (E. Brunetta); IRCCS Ospedale San Raffaele, Milan (L. Dagna); Azienda Sanitaria Universitaria Integrata di Udine, Udine (S. De Vita); Azienda Ospedaliero-Universitaria Careggi, Florence (G. Emmi); AOU Ospedali Riuniti di Ancona, Torrette Ancona (A. Gabrielli); Azienda Ospedaliero Universitaria di Parma, Parma (L. Manenti); ASST di Monza-Ospedale San Gerardo, Monza (F. Pieruzzi); ASL Città di Torino - Ospedale San Giovanni Bosco, Torino (D. Roccatello); Azienda Unità Sanitaria Locale di Reggio Emilia, Reggio Emilia (C. Salvarani).

Japan— National Coordinating Investigator: Prof. M. Harigai, Tokyo Women's Medical University, Tokyo; Kagawa University Hospital, Kagawa (H. Dobashi); Hokkaido University Hospital, Hokkaido (T. Atsumi); University of Miyazaki Hospital, Miyazaki (S. Fujimoto); Teikyo University Chiba Medical Center, Chiba (N. Hagino); National Hospital Organization Yokohama Medical Center, Yokohama (A. Ihata); Kyorin University Hospital, Tokyo (S. Kaname); Keio University Hospital, Tokyo (Y. Kaneko); Juntendo University Shizuoka Hospital, Shizuoka (A. Katagiri); Nagoya Medical Center, Aichi (M. Katayama); Yokohama City University Hospital, Kanagawa (Y. Kirino); National Hospital Organization Kanazawa Medical Center, Ishikawa (K. Kitagawa); Akita University Hospital, Akita City (A. Komatsuda); Teikyo University Hospital, Tokyo (H. Kono); Saitama Medical Center, Saitama (T. Kurasawa); National Hospital Organization Chiba East Hospital, Chiba (R. Matsumura); Saitama Medical University Hospital, Saitama (T. Mimura); Kobe University Hospital, Hyogo (A. Morinobu); Shimane University Hospital, Shimane (Y. Murakawa); Nagoya City University Hospital, Aichi (T. Naniwa); Toho University Omori Medical Center, Tokyo (T. Nanki); Hamamatsu University Hospital, Shizuoka (N. Ogawa); National Hospital Organization Tokyo Medical Center, Tokyo (H. Oshima); Okayama University Hospital, Okayama (K. Sada); Hiroshima University Hospital, Hiroshima (E. Sugiyama); Osaka Medical College Hospital, Osaka (T. Takeuchi); Toyama University Hospital, Toyama (H Taki); Juntendo University Hospital, Tokyo (N. Tamura); Tazuke Kofukai Medical Research Institute Kitano Hospital, Osaka (T. Tsukamoto); University of Tsukuba Hospital, Ibaraki (K. Yamagata); Okayama Saiseikai General Hospital, Okayama (M. Yamamura).

The Netherlands— Erasmus MC, Rotterdam (P. van Daele); Groningen Universitair Medisch Centrum, Groningen (A. Rutgers); Leids Universitair Medisch Centrum, Leiden (Y. Teng).

New Zealand— National Coordinating Center: Dunedin Hospital, Dunedin (R. Walker); Christchurch Clinical Studies Trust, Christchurch (I. Chua); Auckland City Hospital, Auckland (M. Collins); Waikato Hospital, Hamilton (K. Rabindranath); North Shore Hospital, Takpuna, Auckland (J. de Zoysa).

Norway— National Coordinating Center: Akershus Universitetssykehus, Nordbyhagen (M. Svensson); Oslo Universitessykkehus, Oslo (B. Grevbo); University Hospital of North Norway, Tromso (S. Kalstad).

Republic of Ireland— National Coordinating Center: Beaumont Hospital, Dublin (M. Little); Cork University Hospital, Cork (M. Clarkson); St. Vincent's University Hospital, Dublin (E. Molloy).

Spain— Hospital Vall D Hebron, Barcelona (I. Agraz Pamplona); Hospital Sant Joan de Deu, Barcelona (J. Anton); Hospital Universitario Infanta Sofia, San Sebastian de los Reyes, Madrid (V. Barrio Lucia); Hospital Da Costa, Burela (S. Ciggaran); Hospital Clinic Barcelona – Autoimmune Diseases Department, Barcelona (M. Cinta Cid); Fundacio Puigvert, Barcelona (M. Diaz Encarnacion); Hospital Universitari de Bellvitge, Barcelona (X. Fulladosa Oliveras); Hospital del Mar, Barcelona (M. Jose Soler); Hospital Germans Trias i Pujol, Badalona (H. Marco Rusinol); Hospital 12 de Octubre, Madrid (M. Praga); Hospital Clinic Barcelona, Barcelona (L. Quintana Porras); Hospital Universitari Arnau de Vilanova, Lleida (A. Segarra).

Sweden— National Coordinating Center: Karolinska University Hospital, Stockholm (A. Bruchfeld); Linköping University, Linköping (M. Segelmark); Uppsala University Hospital, Uppsala (I. Soveri); Örebro University Hospital, Örebro (E. Thomaidi); Skane University Hospital, Malmo (K. Westman).

Switzerland— National Coordinating Center: Kantonsspital St. Gallen, St. Gallen (T. Neumann); CHUV Lausanne, Lausanne (M. Burnier); University Hospital Basel, Basel (T. Daikeler); Hôpital Fribourgeois, Fribourg (J. Dudler); Immunologie- Zentrum Zürich, Zürich (T. Hauser); Universitätsspital Zürich, Zürich (H. Seeger); Inselspital, Universitätsspital Bern, Bern (B. Vogt).

United Kingdom— National Coordinating Center: Addenbrooke’s Hospital - Cambridge University Hospitals, Cambridge (D. Jayne); Leicester General Hospital, Leicester (J. Burton and R. Al Jayyousi); Leeds Childrens Hospital, Leeds (T. Amin); Leeds Teaching Hospitals NHS Trust, Leeds (J. Andrews); Freeman Hospital, Newcastle upon Tyne (L. Baines); Great Ormond Street Hospital for Children, London (P. Brogan); Southend University Hospital, Westcliff on Sea (B. Dasgupta); Kent and Canterbury Hospital, Canterbury – Kent (T. Doulton); Royal Berkshire Hospital, Reading, Berkshire (O. Flossmann); University Hospital of Wales, Cardiff (S. Griffin); Royal Liverpool University Hospital, Liverpool (J. Harper); University of Birmingham, Birmingham (L. Harper); University Aberdeen, Aberdeen (D. Kidder); Russells Hall Hospital, Dudley (R. Klocke); Queens Medical Centre, Nottingham (P. Lanyon); Nuffield Orthopaedic Centre, Oxford (R. Luqmani); Whytemans Brae Hospital, Fife (J. McLaren); St Helier Hospital, Carshalton (D. Makanjuola); Alder Hey Children's NHS Foundation Trust, Liverpool (L. McCann); Basildon University Hospital, Basildon (A. Nandagudi and S. Selvan); Salford Royal NHS Foundation Trust Manchester, Salford (E. O'Riordan); University of Manchester, Manchester Royal Infirmary, Manchester (M. Patel); Queen Elizabeth University Hospital, Glasgow (R. Patel); Imperial College Healthcare NHS Trust, London (C. Pusey); The Royal London Hospital, London (R. Rajakariar); Bristol Royal Infirmary, Bristol (J. Robson); Guy’s and St Thomas’s NHS Foundation Trust, London (M. Robson); UCL Centre for Nephrology Royal Free, London (A. Salama); Royal Devon and Exeter Hospital, Exeter (L. Smyth); Raigmore Hospital, Inverness (J. Sznajd); Dorset County Hospital, Dorchester (J. Taylor).

United States of America—University of Pennsylvania, Philadelphia (P. Merkel and A. Sreih); Winthrop University Hospital, Mineola (E. Belilos); Columbia University Medical Center, New York (A. Bomback); Virginia Mason Medical Center, Seattle (J. Carlin); University of South Florida, Tampa (Y. Chang Chen Lin); University of North Carolina Hospitals, Chapel Hill (V. Derebail); MedStar Georgetown University Hospital, Washington (S. Dragoi); University of Chicago Medical Center Rheumatology, Chicago (A. Dua); Cedars-Sinai Medical Center, Los Angeles (L. Forbess); Johns Hopkins Bayview Medical Center, Baltimore (D. Geetha); University of Michigan, Ann Arbor (P. Gipson); Rhode Island Hospital, Providence (R. Gohh); Brookview Hills Research Associates, Winston-Salem (G. T. Greenwood); Indiana University Nephrology, Indianapolis (S. Hugenberg); Western Washington Arthritis Clinic, Bothell (R. Jimenez); Northwest Louisiana Nephrology, Shreveport (M. Kaskas); University of California, Los Angeles, Santa Monica (T. Kermani); Altoona Center for Clinical Research, Duncansville (A. Kivitz); University of Utah, Salt Lake City (C. Koening); Cleveland Clinic, Cleveland (C. Langford); Northwell Health, Great Neck (G. Marder); University of Kentucky Medical Center, Lexington (A. Mohamed); Boston University, Boston (P. Monach); Arizona Kidney Disease and Hypertension Center Flagstaff, Flagstaff (N. Neyra); Articularis Healthcare Group, Charleston (G. Niemer); Massachusetts General Hospital, Boston (J. Niles); East Carolina University, Greenville (R. Obi); Renal Disease Research Institute, Dallas (C. Owens); Washington University School of Medicine, St. Louis (D. Parks); Colorado Kidney Care, Denver (A. Podoll); Ohio State University, Columbus (B. Rovin); San Francisco General Hospital Dialysis Center, San Francisco (R. Sam); Rheumatology Associates of North Alabama, Huntsville (W. Shergy); Boise Kidney & Hypertension, PLLC – Meridian, Caldwell (A. Silva); Mayo Clinic - Division of Pulmonary & Critical Care Medicine, Rochester (U. Specks); Hospital for Special Surgery, New York (R. Spiera); University of Kansas Medical Center, Kansas City (J. Springer); University of Colorado Denver - School of Medicine, Aurora (C. Striebich); Arizona Arthritis & Rheumatology Research, Phoenix (A. Swarup); University of Minnesota, Minneapolis (S. Thakar); Emory University School of Medicine, Atlanta (A. Tiliakos); Arthritis, Autoimmune and Allergy LLC, Daytona Beach (Y. Tsai); University of Texas Health Sciences Center, Houston (D. Waguespack); Allegheny General Hospital, Pittsburgh (M. Chester Wasko).
