# Supplementary material for: Efficacy and safety of avacopan in patients aged 65 years and older with ANCA-associated vasculitis: a post hoc analysis of data from the ADVOCATE trial
Source: Rheumatology (Oxford). 2025 Mar 3;64(6):3863–71. doi: 10.1093/rheumatology/keaf122 (PMC12107037; doi:10.1093/rheumatology/keaf122)
Supplement: keaf122_Supplementary_Data [file keaf122_supplementary_data.zip › keaf122_Supplementary_Data/rhe-24-2844-File005.docx]

**Supplementary Table S1: Comorbidities at baseline by system organ class in patients with granulomatosis with polyangiitis or microscopic polyangiitis in the ADVOCATE trial, stratified by age**

| **Comorbidity,  n (%)** | **Age <65 years** | | | **Age 65–74 years** | | | **Age ≥75 years** | | |
| --- | --- | --- | --- | --- | --- | --- | --- | --- | --- |
|  | **Avacopan (n=80)** | **Prednisone taper (n=90)** | **Total  (n=170)** | **Avacopan (n=60)** | **Prednisone taper (n=49)** | **Total  (n=109)** | **Avacopan (n=26)** | **Prednisone taper (n=25)** | **Total  (n=51)** |
| Vascular disorders | 43 (53.8) | 46 (51.1) | 89 (52.4) | 45 (75.0) | 37 (75.5) | 82 (75.2) | 20 (76.9) | 17 (68.0) | 37 (72.5) |
| Metabolism and nutrition disorders | 34 (42.5) | 41 (45.6) | 75 (44.1) | 45 (75.0) | 29 (59.2) | 74 (67.9) | 17 (65.4) | 11 (44.0) | 28 (54.9) |
| Respiratory, thoracic and mediastinal disorders | 45 (56.3) | 63 (70.0) | 108 (63.5) | 42 (70.0) | 28 (57.1) | 70 (64.2) | 17 (65.4) | 19 (76.0) | 36 (70.6) |
| Renal and urinary disorders | 46 (57.5) | 45 (50.0) | 91 (53.5) | 38 (63.3) | 29 (59.2) | 67 (61.5) | 17 (65.4) | 18 (72.0) | 35 (68.6) |
| Musculoskeletal and connective tissue disorders | 47 (58.8) | 55 (61.1) | 102 (60.0) | 39 (65.0) | 25 (51.0) | 64 (58.7) | 14 (53.8) | 15 (60.0) | 29 (56.9) |
| Surgical and medical procedures | 32 (40.0) | 42 (46.7) | 74 (43.5) | 32 (53.3) | 24 (49.0) | 56 (51.4) | 14 (53.8) | 14 (56.0) | 28 (54.9) |
| Infections and infestations | 37 (46.3) | 54 (60.0) | 91 (53.5) | 33 (55.0) | 22 (44.9) | 55 (50.5) | 16 (61.5) | 11 (44.0) | 27 (52.9) |
| Nervous system disorders | 31 (38.8) | 33 (36.7) | 64 (37.6) | 26 (43.3) | 25 (51.0) | 51 (46.8) | 11 (42.3) | 14 (56.0) | 25 (49.0) |
| Blood and lymphatic system disorders | 31 (38.8) | 28 (31.1) | 59 (34.7) | 27 (45.0) | 18 (36.7) | 45 (41.3) | 7 (26.9) | 13 (52.0) | 20 (39.2) |
| Gastrointestinal disorders | 31 (38.8) | 32 (35.6) | 63 (37.1) | 28 (46.7) | 15 (30.6) | 43 (39.4) | 13 (50.0) | 15 (60.0) | 28 (54.9) |
| Social circumstances | 12 (15.0) | 14 (15.6) | 26 (15.3) | 20 (33.3) | 20 (40.8) | 40 (36.7) | 9 (34.6) | 9 (36.0) | 18 (35.3) |
| Investigations | 19 (23.8) | 25 (27.8) | 44 (25.9) | 22 (36.7) | 12 (24.5) | 34 (31.2) | 5 (19.2) | 5 (20.0) | 10 (19.6) |
| General disorders and administration site conditions | 28 (35.0) | 27 (30.0) | 55 (32.4) | 19 (31.7) | 14 (28.6) | 33 (30.3) | 9 (34.6) | 12 (48.0) | 21 (41.2) |
| Cardiac disorders | 15 (18.8) | 16 (17.8) | 31 (18.2) | 20 (33.3) | 11 (22.4) | 31 (28.4) | 6 (23.1) | 8 (32.0) | 14 (27.5) |
| **Comorbidity, n (%)** | **Age <65 years** | | | **Age 65–74 years** | | | **Age ≥75 years** | | |
|  | **Avacopan (n=80)** | **Prednisone taper (n=90)** | **Total  (n=170)** | **Avacopan (n=60)** | **Prednisone taper (n=49)** | **Total  (n=109)** | **Avacopan (n=26)** | **Prednisone taper (n=25)** | **Total  (n=51)** |
| Endocrine disorders | 9 (11.3) | 15 (16.7) | 24 (14.1) | 18 (30.0) | 13 (26.5) | 31 (28.4) | 5 (19.2) | 6 (24.0) | 11 (21.6) |
| Eye disorders | 17 (21.3) | 29 (32.2) | 46 (27.1) | 16 (26.7) | 15 (30.6) | 31 (28.4) | 9 (34.6) | 12 (48.0) | 21 (41.2) |
| Skin and subcutaneous tissue disorders | 23 (28.8) | 37 (41.1) | 60 (35.3) | 17 (28.3) | 13 (26.5) | 30 (27.5) | 7 (26.9) | 3 (12.0) | 10 (19.6) |
| Psychiatric disorders | 23 (28.8) | 26 (28.9) | 49 (28.8) | 19 (31.7) | 10 (20.4) | 29 (26.6) | 10 (38.5) | 7 (28.0) | 17 (33.3) |
| Ear and labyrinth disorders | 22 (27.5) | 27 (30.0) | 49 (28.8) | 15 (25.0) | 13 (26.5) | 28 (25.7) | 9 (34.6) | 7 (28.0) | 16 (31.4) |
| Reproductive system and breast disorders | 10 (12.5) | 12 (13.3) | 22 (12.9) | 17 (28.3) | 6 (12.2) | 23 (21.1) | 9 (34.6) | 6 (24.0) | 15 (29.4) |
| Neoplasms benign, malignant and unspecified | 7 (8.8) | 13 (14.4) | 20 (11.8) | 11 (18.3) | 11 (22.4) | 22 (20.2) | 6 (23.1) | 7 (28.0) | 13 (25.5) |
| Injury, poisoning and procedural complications | 9 (11.3) | 19 (21.1) | 28 (16.5) | 7 (11.7) | 9 (18.4) | 16 (14.7) | 0 (0.0) | 6 (24.0) | 6 (11.8) |
| Immune system disorders | 11 (13.8) | 20 (22.2) | 31 (18.2) | 5 (8.3) | 8 (16.3) | 13 (11.9) | 0 (0.0) | 3 (12.0) | 3 (5.9) |
| Hepatobiliary disorders | 4 (5.0) | 7 (7.8) | 11 (6.5) | 7 (11.7) | 2 (4.1) | 9 (8.3) | 1 (3.8) | 3 (12.0) | 4 (7.8) |
| Congenital, familial and genetic disorders | 4 (5.0) | 4 (4.4) | 8 (4.7) | 5 (8.3) | 3 (6.1) | 8 (7.3) | 0 (0.0) | 0 (0.0) | 0 (0.0) |
| Pregnancy, puerperium and perinatal conditions | 0 (0.0) | 1 (1.1) | 1 (0.6) | 0 (0.0) | 0 (0.0) | 0 (0.0) | 0 (0.0) | 1 (4.0) | 1 (2.0) |
